# Separation of the Teeth

**Published:** 1855-07

**Authors:** J. Taft


### SEPARATION OF THE TEETH.

J. TAFT.

In most cases of approximal decay, the teeth must be separated before filling. Though cases are not unfrequent in which the space between them is sufficient to admit of free manipulation, in the various steps of the operation.

This operation though so important, is very imperfectly performed by many who attempt to fill teeth, and on this account a great number of fillings are less perfect than they would otherwise be. It is a truism, that, in the operation of filling teeth, unless every step in the process is complete, the result will be imperfect. And of no part of the process is it more certainly true than of the separation of the teeth. Unless this first step is thoroughly performed. and space obtained for the introduction and free use of the various instruments required, not any part of the operation can be perfect. Imperfect separation of the teeth is a prominent cause of so many imperfect fillings in approximal cavities. Many imperfect fillings are made, even when ample separation is obtained; yet without it no one can make a good filling.

The objects for which the teeth are separated are various; the most frequent is, to obtain space sufficient in which to operate, with facility, in all parts of the operation of filling. It is sometimes requisite to cut away more than would otherwise be necessary, to remove thin friable edges of the cavity, and to obtain sufficiently firm borders to sustain the filling.

Teeth are sometimes separated for the introduction of clasps: this should never be done if it can be avoided; as it usually proves highly injurious to the teeth. Many of our best operators do not separate teeth at all for this purpose: and this is doubtless the best course. At one time it was a very common practice. It was a general practice at one time to separate the teeth to relieve a crowded condition: this has been abandoned. There are three methods of separating the teeth—with the file, with the chisel or thick cutting instruments, and by pressure. Formerly tbe teeth were separated exclusively with the file, and these of very crude form, and cut: doubtless much injury has been done by the use of such files; especially in unfavorable cases.

When any considerable portion of the dentine is to be removed, the file is not the best instrument. Upon inflamed dentine it is exceedingly painful, it will often increase the inflammation; the jarring is unpleasant to the patient; it is liable to irritate the external periosteum. The action of the file is very tedious; wearisome to the patient and operator. The file is a valuable instrument, the place of which could not be supplied by any other.

When a separation is to be made that requires the removal of a considerable portion of the tooth, the chisels or heavy cutting instruments are preferable to any other. These, if of the proper form and temper, and kept sharp, are very efficient for cutting away portions of the teeth. The execution with them is far more rapid than with the file, and far less unpleasant to the patient. The removal of sensitive dentine can be effected with but little or no pain, and without a liability of increasing the inflammation, or producing irritation or disease of the external periosteum. The force of the instrument is resisted by the entire attachment of the tooth; and the pressure by the instrument is made almost in a line with the axis of the tooth. In the use of these instruments the contiguous teeth do not suffer injury as is often the case with the file.

The manipulation with these instruments is very simple. For separating the front teeth the instrument is firmly held in the hand; and the thumb placed on the points of the teeth, the instrument placed on the teeth at the point from which the portion is to be removed, and pressed gradually toward the gums, cutting its way freely; all effort to thrust it between the teeth as a wedge, or before it has cut its way freely, must be avoided. In this way as much of the dentine can be removed, as is desired, in a few moments. This class of instruments is invaluable for the formation of the V shaped spaces between the bicuspids and molars. It requires a prolonged use of the file to make these separations properly; and hence the practice of attempting to fill the approximal cavities without a separation at all, operating through a small opening at the crown angle of the tooth or through small holes drilled through the outer or inner portion of the tooth. With these instruments, points upon the teeth can be approached, and operated upon with facility, that the file cannot touch. They should be kept sharp or they will be little better than the file. The third method of separation is by pressure. This in many cases is preferable to either of the methods referred to; and especially is this true in regard to the anterior teeth when it is important to preserve the natural form. To make a successful separation in this manner, several considerations must be kept in view. In the first place, there must be sufficient space between the contiguous teeth to permit the desired space to be made. If all the teeth are remaining and stand close together, it will be impossible to obtain much space by pressure, but if there is a slight space between some of the teeth or a neighboring tooth has been removed, the object may readily be obtained.

The gums, periosteum, &c., should be in a healthy condition; much injury may be done by attempting to separate the teeth, when the contiguous parts are in an irritable state. This operation is scarcely admissible in persons of a neuralgic diathesis. When the vital energy is weak, it is scarcely admissible, and particularly when there is an inflammatory tendency. But if attempted at all in such cases it should be very gradual, and with great care. In cases of this kind much may be gained by prior treatment: that should be constitutional rather than local.

There are some cases in which it is best to make the separation by pressure, partly, and then dress off the thin friable edges of the cavity, with the cutting instrument or file, and thus complete the separation. It should be determined before pressure is applied at all, whether the entire separation will be made by that means. Various materials have been used for separating the teeth by pressure. The following are the chief, viz: cotton, wood, india rubber and ligatures. The condition and character of the parts to be operated upon will indicate the material best adapted in any given instance. In a good constitution, the parts healthy, and the teeth firmly set, wood or india rubber may be indicated; but in cases of an opposite character, these should be used very cautiously, or material less active, or, more easily controlled, be used. The amount of pressure to be made, and kept up will be governed by the susceptibility of the parts to irritation. Soreness usually occurs in a few hours after the introduction of the material between the teeth.

The pressure should be gradual and constant. The time necessary for the completion of this process is from ten to twelve days. There should be but one separation made at a time. The pressure is applied slightly at first, and afterwards with more force as the patient will bear it.

The material should be reapplied every day. The teeth should be retained apart until the soreness has abated; and then operate. The process should be continued till ample space is obtained. Teeth that have been separated by pressure if not retained too long, return to their former position. It is supposed by some that separation of the teeth by pressure is admissible only in young persons, or those under thirty years of age. Certainly these are most susceptible; but it is proper under favorable circumstances, thus to separate the teeth of persons of any age.

